# Supplementary material for: Risk and Resilience Variants in the Retinoic Acid Metabolic and Developmental Pathways Associated with Risk of FASD Outcomes
Source: Biomolecules. 2024 May 10;14(5):569. doi: 10.3390/biom14050569 (PMC11117505; doi:10.3390/biom14050569)
Supplement: Supplementary file 1 [file biomolecules-14-00569-s001.zip › Figure S1.pdf]

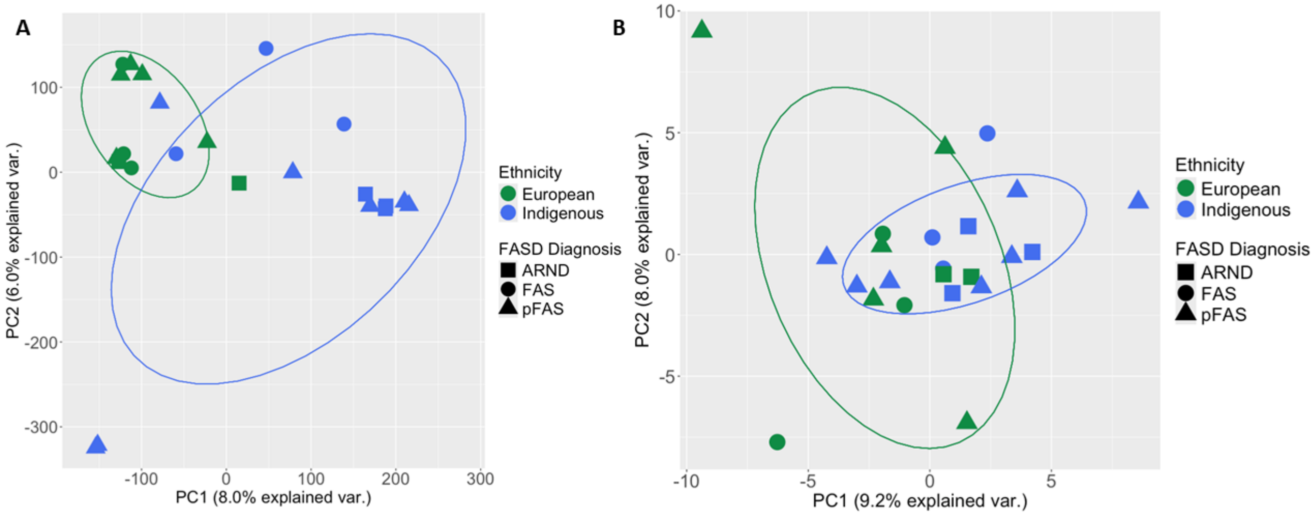

Supplemental Figure S1. PCA analysis of all variants segregate by ethnicity; however, analysis of all variants found significant in this study largely do not. When examining all genetic variants found in the FASD cohort the individuals were found to cluster by ethnicity (Panel A). Genetic variants found to be significant in this study do not separate by ethnicity (Panel B).
