# Supplementary material for: Risk and Resilience Variants in the Retinoic Acid Metabolic and Developmental Pathways Associated with Risk of FASD Outcomes
Source: Biomolecules. 2024 May 10;14(5):569. doi: 10.3390/biom14050569 (PMC11117505; doi:10.3390/biom14050569)
Supplement: Supplementary file 1 [file biomolecules-14-00569-s001.zip › Supplemental Specific References.pdf]

83. Edenberg, H.J. The Genetics of Alcohol Metabolism: Role of Alcohol Dehydrogenase and Aldehyde Dehydrogenase Variants. *Alcohol Res. Health* **2007**, *30*, 5–13.
84. Bierut, L.J.; Goate, A.M.; Breslau, N.; Johnson, E.O.; Bertelsen, S.; Fox, L.; Agrawal, A.; Bucholz, K.K.; Grucza, R.; Hesselbrock, V.; et al. ADH1B Is Associated with Alcohol Dependence and Alcohol Consumption in Populations of European and African Ancestry. *Mol. Psychiatry* **2012**, *5*, 445–450. https://doi.org/10.1038/mp.2011.124.
85. Warren, K.R.; Li, T.K. Genetic Polymorphisms: Impact on the Risk of Fetal Alcohol Spectrum Disorders. *Birth Defects Res. A Clin. Mol. Teratol.* **2005**, *73*, 195–203.
86. Hoang, Y.T.T.; Nguyen, Y.T.; Nguyen, H.D.; Le, A.T.P.; Bui, H.T.T.; Vu, N.P.; Nguyen, H.H. Single Nucleotide Polymorphisms of ADH1B, ADH1C and ALDH2 Genes in 235 People Living in Thai Nguyen Province of Vietnam. *Asian Pac. J. Cancer Prev.* **2022**, *23*, 4243–4251. https://doi.org/10.31557/APJCP.2022.23.12.4243.
87. Luo, X.; Kranzler, H.R.; Zuo, L.; Yang, B.; Lappalainen, J.; Gelernter, J. ADH4 Gene Variation Is Associated with Alcohol and Drug Dependence: Results from Family Controlled and Population-Structured Association Studies. *Pharmacogenet. Genom.* **2005**, *15*, 755–768. https://doi.org/10.1097/01.fpc.0000180141.77036.dc.
88. Luo, X.; Kranzler, H.R.; Zuo, L.; Wang, S.; Gelernter, J. Personality Traits of Agreeableness and Extraversion Are Associated with ADH4 Variation. *Biol. Psychiatry* **2007**, *61*, 599–608. https://doi.org/10.1016/j.biopsych.2006.05.017.
89. Pavan, M.; Ruiz, V.F.; Silva, F.A.; Sobreira, T.J.; Cravo, R.M.; Vasconcelos, M.; Marques, L.P.; Mesquita, S.M.F.; Krieger, J.E.; Lopes, A.A.B.; et al. ALDH1A2 (RALDH2) Genetic Variation in Human Congenital Heart Disease. *BMC Med. Genet.* **2009**, *10*, 113. https://doi.org/10.1186/1471-2350-10-113.
90. Deak, K.L.; Dickerson, M.E.; Linney, E.; Enterline, D.S.; George, T.M.; Melvin, E.C.; Graham, F.L.; Siegel, D.G.; Hammock, P.; Mehltretter, L.; et al. Analysis of ALDH1A2, CYP26A1, CYP26B1, CRABP1, and CRABP2 in Human Neural Tube Defects Suggests a Possible Association with Alleles in ALDH1A2. *Birth Defects Res. A Clin. Mol. Teratol.* **2005**, *73*, 868–875. https://doi.org/10.1002/bdra.20183.
91. Li, K.; Zhong, Y.; Peng, Y.; Zhou, B.; Wang, Y.; Li, Q.; Zhang, Y.; Song, H.; Rao, L. Association Between AXIN1 Gene Polymorphisms and Dilated Cardiomyopathy in a Chinese Han Population. *DNA Cell Biol.* **2019**, *38*, 436–442. https://doi.org/10.1089/dna.2018.4567.
92. Zhou, B.; Tang, T.; Chen, P.; Pu, Y.; Ma, M.; Zhang, D.; Li, L.; Zhang, P.; Song, Y.; Zhang, L. The Variations in the AXIN1 Gene and Susceptibility to Cryptorchidism. *J. Pediatr. Urol.* **2015**, *11*, 132.e1–132.e5. https://doi.org/10.1016/j.jpurol.2015.02.007.
93. Styrkarsdottir, U.; Thorleifsson, G.; Gudjonsson, S.A.; Sigurdsson, A.; Center, J.R.; Lee, S.H.; Nguyen, T.V.; Kwok, T.C.Y.; Lee, J.S.W.; Ho, S.C.; et al. Sequence Variants in the PTCH1 Gene Associate with Spine Bone Mineral Density and Osteoporotic Fractures. *Nat. Commun.* **2016**, *7*, 10129. https://doi.org/10.1038/ncomms10129.
94. Callahan, N.; Modesto, A.; Meira, R.; Seymen, F.; Patir, A.; Vieira, A.R. Axis Inhibition Protein 2 (AXIN2) Polymorphisms and Tooth Agenesis. *Arch. Oral. Biol.* **2009**, *54*, 45–49. https://doi.org/10.1016/j.archoralbio.2008.08.002.
95. Tian, T.; Lei, Y.; Chen, Y.; Karki, M.; Jin, L.; Finnell, R.H.; Wang, L.; Ren, A. Somatic Mutations in Planar Cell Polarity Genes in Neural Tissue from Human Fetuses with Neural Tube Defects. *Hum. Genet.* **2020**, *139*, 1299–1314. https://doi.org/10.1007/s00439-020-02172-0.
96. Parmalee, N.L.; Schubert, C.; Merriam, J.E.; Allikmets, K.; Bird, A.C.; Gillies, M.C.; Peto, T.; Figueroa, M.; Friedlander, M.; Fruttiger, M.; et al. Analysis of Candidate Genes for Macular Telangiectasia Type 2. *Mol. Vis.* **2010**, *16*, 2718–2726.
97. Lasabova, Z.; Stanclova, A.; Grendar, M.; Mikolajcikova, S.; Calkovska, A.; Lenhartova, N.; Ziak, P.; Matasova, K.; Caprnda, M.; Kruzliak, P.; et al. Genetic Association of Single Nucleotide Polymorphisms of FZD4 and BDNF Genes with Retinopathy of Prematurity. *Ophthalmic Genet.* **2018**, *39*, 332–337. https://doi.org/10.1080/13816810.2018.1432064.
98. Magic, M.; Zeljic, K.; Jovandic, S.; Stepic, J.; Pejovic, M.; Colic, S.; Magic, Z.; Supic, G. Hedgehog Signaling Pathway and Vitamin D Receptor Gene Variants as Potential Risk Factors in Odontogenic Cystic Lesions. *Clin. Oral. Investig.* **2018**, *23*, 2675–2684.

99. Chang, J.; Wang, S.; Zheng, Z. Etiology of Hypospadias: A Comparative Review of Genetic Factors and Developmental Processes between Human and Animal Models. *Res. Rep. Urol.* **2020**, *12*, 673–686. https://doi.org/10.2147/RRU.S276141.
100. Renard, E.; Chéry, C.; Oussalah, A.; Josse, T.; Perrin, P.; Tramoy, D.; Voirin, J.; Klein, O.; Leheup, B.; Feillet, F.; et al. Exome Sequencing of Cases with Neural Tube Defects Identifies Candidate Genes Involved in One—Carbon/Vitamin B12 Metabolisms and Sonic Hedgehog Pathway. *Hum. Genet.* **2019**, *138*, 703–713. https://doi.org/10.1007/s00439-019-02015-7.
101. Deneault, E.; Faheem, M.; White, S.H.; Rodrigues, D.C.; Sun, S.; Wei, W.; Piekna, A.; Thompson, T.; Howe, J.L.; Chalil, L.; et al. CNTN5-/+or EHMT2-/+human IPSC-Derived Neurons from Individuals with Autism Develop Hyperactive Neuronal Networks. *Elife* **2019**, *8*, e40092. https://doi.org/10.7554/eLife.40092.
102. Pinard, A.; Eudes, N.; Mitchell, J.; Bajolle, F.; Grelet, M.; Okoronkwo, J.; Bonnet, D.; Collod, G.; Stéphane, B. Analysis of HOXB1 Gene in a Cohort of Patients with Sporadic Ventricular Septal Defect. *Mol. Biol. Rep.* **2018**, *45*, 1507–1513. https://doi.org/10.1007/s11033-018-4212-x.
103. Dargis, N.; Lamontagne, M.; Gaudreault, N.; Sbarra, L.; Henry, C.; Pibarot, P.; Mathieu, P.; Bossé, Y. Identification of Gender-Specific Genetic Variants in Patients With Bicuspid Aortic Valve. *Am. J. Cardiol.* **2016**, *117*, 420–426. https://doi.org/10.1016/j.amjcard.2015.10.058.
104. Girdauskas, E.; Kaemmerer, H.; Kodolitsch, Y. Von Unravelling the Pathogenetic Mechanisms in Congenital Aortopathies: Need for an Integrative Translational Approach. *J. Clin. Med.* **2020**, *9*, 204. https://doi.org/10.3390/jcm9010204.
105. Zhu, C.; Wang, Y.; Zeng, Q.; Qian, Y.; Li, H.; Yang, Z. Combined Effects of Age and Polymorphisms in Notch3 in the Pathogenesis of Cerebral Infarction Disease. *Metab. Brain Dis.* **2016**, *31*, 1157–1164. https://doi.org/10.1007/s11011-016-9868-0.
106. Sowers, L.P.; Loo, L.; Wu, Y.; Campbell, E.; Ulrich, J.D.; Wu, S.; Paemka, L.; Wassink, T.; Meyer, K.; Bing, X.; et al. Disruption of the Non-Canonical Wnt Gene PRICKLE2 Leads to Autism-like Behaviors with Evidence for Hippocampal Synaptic Dysfunction. *Mol. Psychiatry* **2013**, *18*, 1077–1089. https://doi.org/10.1038/mp.2013.71.
107. Koop, A.; Goldmann, P.; Chen, S.R.W.; Thieleczek, R.; Varsa, M. ARVC-Related Mutations in Divergent Region 3 Alter Functional Properties of the Cardiac Ryanodine Receptor. *Biophys. J.* **2008**, *94*, 4668–4677. https://doi.org/10.1529/biophysj.107.122382.
108. Martin, P.; Yang, X.; Robin, N.; Lam, E.; Rabinowitz, J.S.; Erdman, C.A.; Quinn, J.; Weiss, L.A.; Hamilton, S.P.; Kwok, P.; et al. A Rare WNT1 Missense Variant Overrepresented in ASD Leads to Increased Wnt Signal Pathway Activation. *Transl. Psychiatry* **2013**, *3*, e301. https://doi.org/10.1038/tp.2013.75.
109. Christodoulides, C.; Scarda, A.; Granzotto, M.; Milan, G.; Dalla Nora, E.; Keogh, J.; De Pergola, G.; Stirling, H.; Pannacciulli, N.; Sethi, J.; et al. WNT10B Mutations in Human Obesity. *Diabetologia* **2006**, *49*, 678–684. https://doi.org/10.1007/s00125-006-0144-4.WNT10B.
110. Mues, G.; Bonds, J.; Xiang, L.; Vieira, A.R.; Seymen, F.; Klein, O.; Souza, R.N.D. The WNT10A Gene in Ectodermal Dysplasias and Selective Tooth Agenesis. *Am. J. Med. Genet. A* **2014**, *164*, 2455–2460. https://doi.org/10.1002/ajmg.a.36520.
111. Huang, Y.; Jiang, L.; Yang, H.; Wu, L.; Xu, N.; Zhou, X.; Li, J. Variations of Wnt/B-catenin Pathway-related Genes in Susceptibility to Knee Osteoarthritis: A Three-centre Case-control Study. *J. Cell Mol. Med.* **2019**, *23*, 8246–8257. https://doi.org/10.1111/jcmm.14696.
112. Li, Y.; Liu, H.; Dong, Y. Significance of Neurexin and Neuroligin Polymorphisms in Regulating Risk of Hirschsprung's Disease. *J. Investig. Med.* **2018**, *66*, 944–951. https://doi.org/10.1136/jim-2017-000623.
113. Corsten-Janssen, N.; Saitta, S.C.; Hoefsloot, L.H.; McDonal-McGinn, D.M.; Driscoll, D.A.; Derks, R.; Dickinson, K.A.; Kerstjens-Frederiske, W.S.; Emanuel, B.S.; Zackai, E.H.; et al. More Clinical Overlap between 22q11 . 2 Deletion Syndrome and CHARGE Syndrome than Often Anticipated. *Mol. Syndromol.* **2013**, *4*, 235–245. https://doi.org/10.1159/000351127.
114. Cuola, D.; Li, Y.; Yuanyuan, C.; Jianghua, L.; Shuguang, W. Association Study of Catechol-o-Methyltransferase and Alpha-1-Adrenergic Receptor Gene Polymorphisms with Multiple Phenotypes of Heroin Use Disorder. *Neurosci. Lett.* **2021**, *748*, 135677. https://doi.org/10.1016/j.neulet.2021.135677.

115. Bender, H.-U.; Almashanu, S.; Steel, G.; Hu, C.-A.; Lin, W.-W.; Willis, A.; Pulver, A.; Valle, D. Functional Consequences of PRODH Missense Mutations. *Am. J. Hum. Genet.* **2005**, *76*, 409–420.
116. Clelland, C.L.; Drouet, V.; Rilett, K.C.; Smeed, J.A.; Nadrich, R.H.; Rajparia, A.; Read, L.L.; Clelland, J.D. Evidence That COMT Genotype and Proline Interact on Negative-Symptom Outcomes in Schizophrenia and Bipolar Disorder. *Transl. Psychiatry* **2016**, *6*, e891. https://doi.org/10.1038/tp.2016.157.
117. Mozzi, A.; Guerini, F.R.; Forni, D.; Costa, A.S.; Nemni, R.; Baglio, F.; Cabinio, M.; Riva, S.; Pontremoli, C.; Clerici, M.; et al. REST, a Master Regulator of Neurogenesis, Evolved under Strong Positive Selection in Humans and in Non Human Primates. *Sci. Rep.* **2017**, *7*, 9530. https://doi.org/10.1038/s41598-017-10245-w.
118. Ravizza, T.; Onat, F.Y.; Brooks-Kayal, A.R.; Depaulis, A.; Galanopoulou, A.S.; Mazarati, A.; Numis, A.L.; Sankar, R.; Friedman, A. WONOEP Appraisal: Biomarkers of Epilepsy-Associated Comorbidities. *Epilepsia* **2017**, *58*, 331–342. https://doi.org/10.1111/epi.13652.WONOEP.
119. Xu, Y.; Wang, J.; Xu, R.; Zhao, P.; Wang, X.; Sun, H.; Bao, L.; Shen, J. Detecting 22q11.2 Deletion in Chinese Children with Conotruncal Heart Defects and Single Nucleotide Polymorphisms in the Haploid TBX1 Locus. *BMC Med. Genet.* **2011**, *12*, 169. https://doi.org/10.1186/1471-2350-12-169.
120. Akiyama, K.; Saito, A.; Saito, S.; Ozeki, Y.; Watanabe, T. Association of Genetic Variants at 22q11.2 Chromosomal Region with Cognitive Performance in Japanese Patients with Schizophrenia. *Schizophr. Res. Cogn.* **2019**, *17*, 100134. https://doi.org/10.1016/j.scog.2019.100134.
121. Caputo, V.; Strafella, C.; Termine, A.; Campione, E. RNAseq-Based Prioritization Revealed COL6A5, COL8A1, COL10A1 and MIR146A as Common and Differential Susceptibility Biomarkers for Psoriasis and Psoriatic Arthritis: Confirmation from Genotyping Analysis of 1417 Italian Subjects. *Int. J. Mol. Sci.* **2020**, *21*, 2740.
122. Den Hollander, W.; Pulyakhina, I.; Boer, C.; Bomer, N.; Van Der Breggen, R.; Arindrarto, W.; De Almeida, R.C.; Lakenberg, N.; Sentner, T.; Laros, J.F.J.; et al. Annotating Transcriptional Effects of Genetic Variants in Disease-Relevant Tissue: Transcriptome-Wide Allelic Imbalance in Osteoarthritic Cartilage. *Arthritis Rheumatol.* **2019**, *71*, 561–570. https://doi.org/10.1002/art.40748.
123. Trefilova, V.V.; Shnayder, N.A.; Petrova, M.M.; Kaskaeva, D.S.; Tutynina, O.V.; Petrov, K.V.; Popova, T.E.; Balberova, O.V.; Medvedev, G.V.; Nasyrova, R.F. The Role of Polymorphisms in Collagen-Encoding Genes in Intervertebral Disc Degeneration. *Biomolecules* **2021**, *11*, 1279.
124. Jamieson, S.E.; De Roubaix, L.; Cortina-borja, M.; Tan, H.K.; Mui, E.J.; Cordell, H.J.; Kirisits, M.J.; Miller, E.N.; Peacock, C.S.; Hargrave, A.C.; et al. Genetic and Epigenetic Factors at COL2A1 and ABCA4 Influence Clinical Outcome in Congenital Toxoplasmosis. *Investig. Ophthalmol. Vis. Sci.* **2008**, *3*, e2285. https://doi.org/10.1371/journal.pone.0002285.
125. Tang, Y.; Epstein, M.P.; Anderson, G.M.; Zabetian, C.P.; Cubells, J.F. Genotypic and Haplotypic Associations of the DBH Gene with Plasma Dopamine b -Hydroxylase Activity in African Americans. *Eur. J. Hum. Genet.* **2007**, *15*, 878–883. https://doi.org/10.1038/sj.ejhg.5201838.
126. Gonzalez-Lopez, E.; Kawasawa-imamura, Y.; Zhang, L.; Huang, X.; Koltun, W.A.; Coates, M.D.; Vrana, K.E. A Single Nucleotide Polymorphism in Dopamine Beta Hydroxylase (Rs6271 (C > T)) Is over-Represented in Inflammatory Bowel Disease Patients and Reduces Circulating Enzyme. *PLoS ONE* **2019**, *14*, e0210175. https://doi.org/10.1371/journal.pone.0210175.
127. Punchaichira, T.J.; Dey, S.K.; Mukhopadhyay, A. Characterization of SNPs in the Dopamine-β-Hydroxylase Gene Providing New Insights into Its Structure-Function Relationship. *Neurogenetics* **2017**, *18*, 155–168. https://doi.org/10.1007/s10048-017-0519-3.
128. Ates, O.; Cam, F.; Erdogan, S.; Sezer, S.; Karakus, N. Association between 1603C > T Polymorphism of DBH Gene and Bipolar Disorder in a Turkish Population. *Gene* **2013**, *519*, 356–359. https://doi.org/10.1016/j.gene.2013.01.031.
129. Kukshal, P.; Chowdari, V.; Srivastava, V.; Wood, J.; Mcclain, L.; Bhatia, T.; Bhagwat, A.M.; Neelkanth, S.; Laxmikant, V.; Thelma, B.K. Dopaminergic Gene Polymorphisms and Cognitive Function in a North Indian Schizophrenia Cohort. *J. Psychiatr. Res.* **2013**, *47*, 1615–1622. https://doi.org/10.1016/j.jpsychires.2013.07.007.
130. Yeung, E.W.; Craggs, J.G.; Gizer, I.R. Comorbidity of Alcohol Use Disorder and Chronic Pain: Genetic Influences on Brain Reward and Stress Systems. *Alcohol. Clin. Exp. Res.* **2017**, *41*, 1831–1848. https://doi.org/10.1111/acer.13491.

131. Meyers, J.L.; Nyman, E.; Loukola, A.; Rose, R.J.; Kaprio, J.; Dick, D.M. The Association between DRD2/ANKK1 and Genetically Informed Measures of Alcohol Use and Problems. *Addict. Biol.* **2013**, *18*, 523–536. https://doi.org/10.1111/j.1369-1600.2012.00490.x.
132. Franco, G.B.; Mara, F.; Bertoli, D.P.; De Souza, J.F.; Scariot, R. Dopamine Receptor D2 and Ankyrin Repeat Domain Containing One in Temporomandibular Disorder of Adolescents. *Int. J. Paediatr. Dent.* **2019**, *29*, 748–755. https://doi.org/10.1111/ipd.12544.
133. Popescu, A.; Marian, M.; Drăgoi, A.N.A.M.; Costea, R.V. Understanding the Genetics and Neurobiological Pathways behind Addiction (Review). *Exp. Ther. Med.* **2021**, *21*, 544. https://doi.org/10.3892/etm.2021.9976.
134. Lundwall, R.A.; Guo, D.; Dannemiller, J.L. Exogenous Visual Orienting Is Associated with Specific Neurotransmitter Genetic Markers: A Population-Based Genetic Association Study. *PLoS ONE* **2012**, *7*, e30731. https://doi.org/10.1371/journal.pone.0030731.
135. Kirley, A.; Lowe, N.; Mullins, C.; Mccarron, M.; Daly, G.; Waldman, I.; Fitzgerald, M.; Gill, M.; Hawi, Z. Phenotype Studies of the DRD4 Gene Polymorphisms in ADHD: Association With Oppositional Defiant Disorder and Positive Family History Analysis of Association With. *Am. J. Med. Genet. Part B* **2004**, *131*, 38–42. https://doi.org/10.1002/ajmg.b.30072.
136. Nemoda, Z.; Szekely, A.; Sasvari-szekely, M. Neuroscience and Biobehavioral Reviews Psychopathological Aspects of Dopaminergic Gene Polymorphisms in Adolescence and Young Adulthood. *Neurosci. Biobehav. Rev.* **2011**, *35*, 1665–1686. https://doi.org/10.1016/j.neubiorev.2011.04.002.
137. Nakajima, M.; Hattori, E.; Yamada, K.; Iwayama, Y.; Toyota, T.; Iwata, Y.; Tsuchiya, K.; Sugihara, G.; Hashimoto, K.; Watanabe, H.; et al. Association and Synergistic Interaction between Promoter Variants of the DRD4 Gene in Japanese Schizophrenics. *J. Hum. Genet.* **2007**, *52*, 86–91. https://doi.org/10.1007/s10038-006-0084-3.
138. Wang, S.; Chen, Y.; Lee, C.; Cheng, C. Opioid Addiction, Genetic Susceptibility, and Medical Treatments: A Review. *Int. J. Mol. Sci.* **2019**, *20*, 4294. https://doi.org/10.3390/ijms20174294.
139. Haenisch, B.; Linsel, K.; Br, M.; Gilsbach, R.; Propping, P.; Markus, M.N.; Rietschel, M.; Fimmers, R.; Maier, W.; Zobel, A.; et al. Association of Major Depression With Rare Functional Variants in Norepinephrine Transporter and Serotonin 1A Receptor Genes. *Am. J. Med. Genet. Part B* **2008**, *150*, 1013–1016. https://doi.org/10.1002/ajmg.b.30912.
140. Brüss, M.; Kostanian, A.; Bönisch, H.; Göthert, M. The Naturally Occurring Arg 219 Leu Variant of the Human 5-HT 1A Receptor: Impairment of Signal Transduction. *Pharmacog. Genom.* **2005**, *15*, 257–264. https://doi.org/10.1097/01213011-200504000-00009.
141. Fanous, A.H.; Chen, X.; Wang, X.; Amdur, R.; O′Neill, A.; Walsh, D.; Kendler, K. Genetic Variation in the Serotonin 2A Receptor and Suicidal Ideation in a Sample of 270 Irish High-Density Schizophrenia Families. *Am. J. Med. Genet. B Neuropsychiatr. Genet.* **2009**, *150*, 411–417. https://doi.org/10.1002/ajmg.b.30833.
142. Tavares, G.A.; Torres, A.; Souza, J.A. De Early Life Stress and the Onset of Obesity : Proof of MicroRNAs ' Involvement Through Modulation of Serotonin and Dopamine Systems' Homeostasis. *Front. Physiol.* **2020**, *11*, 925. https://doi.org/10.3389/fphys.2020.00925.
143. Mccaffery, J.M.; Duan, Q.L.; Frasure-Smith, N.; Lespérance, F.; Théroux, P.; Rouleau, G.A.; Dubé, M.-P. Genetic Predictors of Depressive Symptoms in Cardiac Patients. *Am. J. Med. Genet. B Neuropsychiatr. Genet.* **2009**, *150*, 381–388. https://doi.org/10.1002/ajmg.b.30824.
144. Jakubczyk, A.; Klimkiewicz, A.; Kopera, M.; Krasowska, A.; Wrzosek, M.; Matsumoto, H.; Burmeister, M.; Brower, K.J.; Wojnar, M. The CC Genotype in the T102C HTR2A Polymorphism Predicts Relapse in Individuals after Alcohol Treatment. *J. Psychiatr. Res.* **2013**, *47*, 527–533. https://doi.org/10.1016/j.jpsychires.2012.12.004.
145. Jakubczyk, A.; Wrzosek, M.; Łukaszkiewicz, J.; Sadowska-Mazuryk, J.; Matsumoto, H.; Śliwerska, E.; Glass, J.; Burmeister, M.; Browser, K.J.; Wojnar, M. The CC Genotype in HTR2A T102C Polymorphism Is Associated with Behavioral Impulsivity in Alcohol-Dependent Patients. *J. Psychiatr. Res.* **2012**, *46*, 44–49. https://doi.org/10.1016/j.jpsychires.2011.09.001.
146. Cao, J.; Liu, X.; Han, S.; Zhang, C.K.; Liu, Z.; Li, D. Association of the HTR2A Gene with Alcohol and Heroin Abuse. *Hum. Genet.* **2014**, *133*, 357–365. https://doi.org/10.1007/s00439-013-1388-y.

147. Sinopoli, V.M.; Burton, C.L.; Arnold, P.D. Neuroscience and Biobehavioral Reviews Review Article A Review of the Role of Serotonin System Genes in Obsessive-Compulsive Disorder. *Neurosci. Biobehav. Rev.* **2017**, *80*, 372–381. https://doi.org/10.1016/j.neubiorev.2017.05.029.
148. Cheah, S.-Y.; Lawford, B.R.; Young, R.M.; Morris, C.P.; Voisey, J. MRNA Expression and DNA Methylation Analysis of Serotonin Receptor 2A (HTR2A) in the Human Schizophrenic Brain. *Genes* **2017**, *8*, 14. https://doi.org/10.3390/genes8010014.
149. Rappold, G.; Walstab, J.; Hammer, C.; Bo, H. Naturally Occurring Variants in the HTR3B Gene Significantly Alter Properties of Human Heteromeric 5-Hydroxytryptamine-3A/B Receptors. *Pharmacog. Genom.* **2008**, *18*, 793–802. https://doi.org/10.1097/FPC.0b013e3283050117.
150. Yosifova, A.; Mushiroda, T.; Stoianov, D.; Vazharova, R.; Dimova, I.; Karachanak, S.; Zaharieva, I.; Milanova, V.; Madjirova, N.; Gerdjikov, I.; et al. Case-Control Association Study of 65 Candidate Genes Revealed a Possible Association of a SNP of HTR5A to Be a Factor Susceptible to Bipolar Disease in Bulgarian Population. *J. Affect. Disord.* **2009**, *117*, 87–97. https://doi.org/10.1016/j.jad.2008.12.021.
151. Stoychev, K.; Dilkov, D.; Naghavi, E.; Kamburova, Z. Genetic Basis of Dual Diagnosis: A Review of Genome-Wide Association Studies (GWAS) Focusing on Patients with Mood or Anxiety Disorders and Co-Occurring Alcohol-Use Disorders. *Diagnostics* **2021**, *11*, 1055. https://doi.org/10.3390/diagnostics11061055.
152. Birkett, J.T.; Arranz, M.J.; Munro, J.; Osbourn, S.; Kerwin, R.W.; Collier, D.A. Association Analysis of the 5-HT5A Gene in Depression, Psychosis and Antipsychotic Response. *Neuroreport* **2000**, *11*, 2017–2020. https://doi.org/10.1097/00001756-200006260-00042.
