# Supplementary material for: Risk and Resilience Variants in the Retinoic Acid Metabolic and Developmental Pathways Associated with Risk of FASD Outcomes
Source: Biomolecules. 2024 May 10;14(5):569. doi: 10.3390/biom14050569 (PMC11117505; doi:10.3390/biom14050569)
Supplement: Supplementary file 1 [file biomolecules-14-00569-s001.zip › Supplemental Table S1 Retinoic Acid and Ethanol Metabolism Gene Candidate List.pdf]

Supplemental Table S1. Retinoic Acid and Ethanol Metabolism Gene Candidate List.

Gene	Biological Role of Gene
ADH1B	Converts ethanol to acetaldehyde and retinol to RA
ADH1C	Converts ethanol to acetaldehyde and retinol to RA
ADH4	Converts ethanol to acetaldehyde and retinol to RA
ADH7	Converts ethanol to acetaldehyde and retinol to RA
ADHFE1	Converts ethanol to acetaldehyde and retinol to RA
ALDH1/1	Acetaldehyde to acetic acid and retinaldehyde to RA
ALDH1/2	Acetaldehyde to acetic acid and retinaldehyde to RA
ALDH2A1	Acetaldehyde to acetic acid and retinaldehyde to RA
ALDH2A2	Acetaldehyde to acetic acid and retinaldehyde to RA
ALDHA3	Acetaldehyde to acetic acid and retinaldehyde to RA
BCO1	Cleaves β-carotene to form two retinal molecules
BCO2	Cleaves β-carotene to form apocarotenoids
BCO2L	Cleaves β-carotene to form apocarotenoids
CES3	Esterification of retinyl esters
CRAB1	Transports retinoic acid in the cell
CRAB2	Transports retinoic acid in the cell
CRBP	Mediates both retinol esterification to retinyl esters and retinol oxidation to retinal and retinoic acid.
CYP26A1	Degradation of retinoic acid
CYP26B1	Degradation of retinoic acid
CYP26C1	Degradation of retinoic acid
DGAT1	Esterification of retinol esters
DHRS3	Reduction of retinaldehyde to retinol
DHRS9	Reduction of retinaldehyde to retinol
FABP3	Mobilization of retinoic acid
FABP4	Mobilization of retinoic acid
LIPC	Mobilization of retinoic acid
LRAT	Creates retinyl esters
NCOR1	Represses retinoic acid mediated transcription
NCOR2	Represses retinoic acid mediated transcription
PNLIP	Creates retinol from retinyl esters
PNPLA4	Creates retinol from retinyl esters
RARA	Retinoic acid mediated transcription factor
RARB	Retinoic acid mediated transcription factor
RARG	Retinoic acid mediated transcription factor
RBP4	Transport of retinol
RDH11	Transport of retinol
RDH13	Transport of retinol
RDH14	Transport of retinol
RDH16	Transport of retinol
RDH5	Retinoic acid mediated transcription factor
RXRA	Retinoic acid mediated transcription factor
RXRB	Retinoic acid mediated transcription factor

RXRG	Retinoic acid mediated transcription factor
SCARB1	Creates retinaldehyde from β-carotene
SDR16C5	Converts retinol into retinaldehyde
STRA6	Transports retinol into the cell
