# Supplementary material for: Risk and Resilience Variants in the Retinoic Acid Metabolic and Developmental Pathways Associated with Risk of FASD Outcomes
Source: Biomolecules. 2024 May 10;14(5):569. doi: 10.3390/biom14050569 (PMC11117505; doi:10.3390/biom14050569)
Supplement: Supplementary file 1 [file biomolecules-14-00569-s001.zip › Supplemental Table S2 Retinoic Acid-Dependent Signaling Network Candidate Genes.pdf]

Supplemental Table S2. Retinoic Acid-Dependent Signaling Network Candidate Genes.

Gene	Signaling Pathway Involved
ABCA1	Cholesterol Metabolism/SHH
ADAMTSL2	TGF-Beta protein storage
ADDUCIN	Rho Kinase Signaling
APOE	Cholesterol Metabolism/SHH
AXIN1	WNT Signaling
AXIN2	WNT Signaling
BMP2	SMAD Signaling
BMP4	SMAD Signaling
CAMK genes	Ca^{2+} Dependent Signaling
CAV genes	ERK/Cholesterol Dependent Signaling
CELSR	PCP WNT Signaling
CTNNB1	Canonical WNT Signaling
CYP51	Cholesterol Metabolism/SHH
DAAM genes	Wnt signaling
DVL genes	WNT signaling
EGF	MAPK/EGF
EGFR genes	MAPK/EGF
ENOS genes	Rho Kinase Signaling
EPAC genes	Rho Kinase Signaling
FDFT1	Cholesterol Metabolism/SHH
FDPS	Cholesterol Metabolism/SHH
FGF8	MAPK/FGF
FGFR genes	MAPK/FGF
FZD genes	Wnt signaling
GLI1	SHH pathway
GLI2	SHH pathway
GLI3	SHH pathway
GSK3B	WNT signaling
HMGCR	Cholesterol Metabolism/SHH
HOXA1	Hox Signaling
HOXA2	Hox Signaling
HOXB1	Hox Signaling
HOXB2	Hox Signaling
IDI1	Cholesterol Metabolism/SHH
IHH	SHH signaling
INSIG1	Cholesterol Metabolism/SHH
INSIG2	Cholesterol Metabolism/SHH
L1CAM	RasMAPK Signaling
LSS	Cholesterol Metabolism/SHH
MAP2K1	RasMAPK Signaling
MAP2K2	RasMAPK Signaling
MVD	Cholesterol Metabolism/SHH

MVK	Cholesterol Metabolism/SHH
NODAL	TGF-Beta signaling
NOTCH genes	NOTCH signaling
NSDHL	Cholesterol Metabolism/SHH
PEX11B	Cholesterol Metabolism/SHH
PEX14	Cholesterol Metabolism/SHH
PEX19	Cholesterol Metabolism/SHH
PI4KA	Cholesterol Metabolism/SHH
PKC	Calcium Signaling
PMVK	Cholesterol Metabolism/SHH
PRICKLE genes	WNT PCP signaling
PTCH genes	SHH pathway
RAC	Cholesterol Metabolism/SHH
RAP1	Cholesterol Metabolism/SHH
RHO kinase genes	Cholesterol Metabolism/SHH
ROCK genes	Cholesterol Metabolism/SHH
RYR genes	Cholesterol Metabolism/SHH
SFRP genes	Calcium Signaling
SHH	Cholesterol Metabolism/SHH
SMO	WNT PCP signaling
SOX10	SHH pathway
SQLE	Cholesterol Metabolism/SHH
SUFU	Cholesterol Metabolism/SHH
NODAL	Cholesterol Metabolism/SHH
NOTCH genes	WNT PCP signaling
NSDHL	SHH pathway
PI4KA	Cholesterol Metabolism/SHH
PKC	Cholesterol Metabolism/SHH
PMVK	Calcium Signaling
PRICKLE genes	Cholesterol Metabolism/SHH
PTCH genes	WNT PCP signaling
RAC	Rho Kinase Signaling
RAP1	Rho Kinase Signaling
RHO kinase genes	Rho Kinase Signaling
ROCK genes	Rho Kinase Signaling
RYR genes	Calcium Signaling
SFRP genes	WNT signaling
SHH	SHH pathway
SMO	Cholesterol Metabolism/SHH
SOX10	TGF-beta, WNT
SQLE	Cholesterol Metabolism/SHH
SUFU	SHH signaling
TAU genes	Rho Kinase Signaling
TGFBR2	SMAD Signaling
VANGL genes	WNT PCP signaling
WNT genes	WNT
