# Supplementary material for: Risk and Resilience Variants in the Retinoic Acid Metabolic and Developmental Pathways Associated with Risk of FASD Outcomes
Source: Biomolecules. 2024 May 10;14(5):569. doi: 10.3390/biom14050569 (PMC11117505; doi:10.3390/biom14050569)
Supplement: Supplementary file 1 [file biomolecules-14-00569-s001.zip › Supplemental Table S3 Candidate Genes in the Causative Genes of Rare Neurodevelopmental Disorders and their Interactive Partners.pdf]

Supplemental Table S3. Candidate Genes in the Causative Genes of Rare Neurodevelopmental Disorders and their Interactive Partners.

Gene	Neurodevelopmental Disorder
A2ML1	Noonan Syndrome gene
AIFM3	22q11.2DS gene
ANKRD11	Cornelia de Lange Syndrome gene
ARVCF	22q11.2DS gene
B3GLCT	Peter Plus Syndrome Gene
BMI-1	interacts with CHD7
BRAF	Noonan Syndrome gene
BRD4	Cornelia de Lange Syndrome gene
BRG1	Cornelia de Lange Syndrome interaction partner
CBL genes	Noonan Syndrome gene
CCDC188	22q11.2DS gene
CCDC8	Three M Syndrome 2 Gene
CDC42	Aarskog interaction partner
CDC45	22q11.2DS gene
CDK8	Opitz-Kaveggia Syndrome interaction partner
CHD7	CHARGE Syndrome gene
CCDC8	Three M Syndrome 1 gene
CLDN5	22q11.2DS gene
CLTCL1	22q11.2DS gene
COMT	22q11.2DS gene
CRKL	22q11.2DS gene
CRMP2	Aarskog and Noonan Syndromes interaction
CUL7	Three M Syndrome 2 Gene
DGCR14	22q11.2DS gene
DGCR2	22q11.2DS gene
DGCR6	22q11.2DS gene
DGCR6L	22q11.2DS gene
DGCR8	22q11.2DS gene
DHCR7	Smith-Lemli-Optiz Syndrome
DUSP4	interacts with CHD7
FGD1	Aarskog Syndrome gene
FGFR2	MAPK/FGF/Apert Syndrome gene
GNB1L	22q11.2DS gene
GP1BB	22q11.2DS gene
GSC2	22q11.2DS gene
HDAC8	Cornelia de Lange Syndrome gene
HIC2	22q11.2DS gene
HIRA	22q11.2DS gene
KLHL22	22q11.2DS gene

KMT2D	Kabuki Syndrome gene
KRAS	Noonan Syndrome gene
LINC00896	22q11.2DS gene
LOC101927859	22q11.2DS gene
LRRC748	22q11.2DS gene
LZTR1	22q11.2DS gene/Noonan Syndrome gene
MAU2	Cornelia de Lange Syndrome interaction
MED12	Opitz-Kaveggia Syndrome gene
MED13	Opitz-Kaveggia Syndrome interaction partner
MED15	Opitz-Kaveggia Syndrome interaction partner and 22q11.2DS gene
MID1	Opitz G/BBB Syndrome gene
mir1286	22q11.2DS gene
mir185	22q11.2DS gene
mir3618	22q11.2DS gene
mir4761	22q11.2DS gene
mir649	22q11.2DS gene
mir6816	22q11.2DS gene
MRPL40	22q11.2DS gene
NIPBL	Cornelia de Lange Syndrome gene
NRAS	Noonan Syndrome gene
OBSL1	3M Syndrome type 2 Syndrome gene
P2RX6	22q11.2DS gene
PARQ3B	CHARGE Syndrome interaction partner
PRODH	22q11.2DS gene
PTPN11	Noonan Syndrome gene
RAD21	Cornelia de Lange Syndrome gene
RANBP1	22q11.2DS and Noonan Syndrome gene
RECQL3	Bloom Syndrome gene
REST	Opitz-Kaveggia Syndrome interaction partner
RIT1	Noonan Syndrome gene
RTN4R	22q11.2DS gene
SCARF2	22q11.2DS gene
SCRAP	Floating-Harbour Syndrome gene
SEPT5	22q11.2DS gene
SERPIND1	22q11.2DS gene
SHOC2	Noonan Syndrome gene
SLC25A1	22q11.2DS gene
SLC7A4	22q11.2DS gene
SMC1A	Cornelia de Lange Syndrome gene
SMC3	Cornelia de Lange Syndrome gene
SNAP29	22q11.2DS gene
SOS1	Noonan Syndrome gene
SOS2	Noonan Syndrome gene
SOX9	Campomelic Dysplasia gene

SPECC1L	Opitz G/BBB Syndrome gene
TANGO2	22q11.2DS gene
TBX1	22q11.2DS gene
THAP7	22q11.2DS gene
TRMT2A	22q11.2DS gene
TSSK2	22q11.2DS gene
TXNRD2	22q11.2DS gene
UFD1L	22q11.2DS gene
USP41	22q11.2DS gene
ZDHHC8	22q11.2DS gene
ZNF74	22q11.2DS gene
