# Supplementary material for: Risk and Resilience Variants in the Retinoic Acid Metabolic and Developmental Pathways Associated with Risk of FASD Outcomes
Source: Biomolecules. 2024 May 10;14(5):569. doi: 10.3390/biom14050569 (PMC11117505; doi:10.3390/biom14050569)
Supplement: Supplementary file 1 [file biomolecules-14-00569-s001.zip › Supplemental Table S4 Candidate Genes in Neurotransmitter, Forebrain Development and Collagen Genes.pdf]

Supplemental Table S4. Candidate Genes in Neurotransmitter, Forebrain Development and Collagen Genes.

Gene	Signaling Pathway Involved
COL genes	Structural Support Proteins
DBH	Dopamine
DRD genes	Dopamine
HTR genes	Serotonin
MAO	Dopamine/Serotonin
OTP	Forebrain Development
OXT	Oxytocin Signaling
OXTR	Oxytocin Receptor
SIM1	Forebrain Development
TH	Dopamine/Serotonin
