# Supplementary material for: Risk and Resilience Variants in the Retinoic Acid Metabolic and Developmental Pathways Associated with Risk of FASD Outcomes
Source: Biomolecules. 2024 May 10;14(5):569. doi: 10.3390/biom14050569 (PMC11117505; doi:10.3390/biom14050569)
Supplement: Supplementary file 1 [file biomolecules-14-00569-s001.zip › Supplemental Table S5 Variant Analysis Results of Retinoic Acid and Alcohol Metabolism Candidate - Revised.pdf]

**Supplemental Table S5.** Variant Analysis Results of Retinoic Acid and Alcohol Metabolism Candidate Genes.

| rsID | Gene | Type of Mutation | Ref Allele | Presump-tive Risk Allele | Biological Impact of Variant | Ref. | FASD Cohort Risk Allele Count Frequency | Thousand Genomes Risk Allele Count (Frequency) | PolyPhen / Sift Prediction | CADD PHRED Score | Statistic | *p*-Value | Passed Benjamini -Hochberg Threshold | Risk or Resilience |
|---|---|---|---|---|---|---|---|---|---|---|---|---|---|---|
| rs1229984 | *ADH1B* | Missense | T | C | C allele associated with higher levels of alcohol consumption and FASD | [32,36, 83] | 45/46 (0.96) | 8428/10016 (0.82) | Probably Damaging / Tolerated | 14.83 | 8.2 | $1.70\times10^{-2}$ | No | Risk * |
| rs113075608 | *ADH1B* | Missense | A | G | Variant has damaging PolyPhen score and Sift Score | - | 1/46 (0.02) | 50/10016 ($5\times10^{-3}$) | Damaging / Damaging | 23.8 | 97.8 | $5.83\times10^{-22}$ | Yes | Risk * |
| rs2066702 | *ADH1B* | Missense | G | G | A allele is noted to be protective against PAE outcomes | [32,85] | 46/46 (1) | 9484/10016 (0.95) | NA / NA | 26.3 | 10.55 | $5.00\times10^{-3}$ | Yes | Risk * |
| rs1612735 | *ADH1C* | Intron | T | C | C allele is associated with alcohol dependence | [72] | 24/56 (0.52) | 2148/10016 (0.21) | NA / NA | 7.85 | 26.5 | $1.77\times10^{-6}$ | Yes | Risk |
| rs698 | *ADH1C* | Missense | T | C | C variant 2-fold less kinetically active and associated with alcohol dependence and use | [38,86] | 25/46 (0.54) | 2146/10016 (0.21) | NA / NA | 14.59 | 30.28 | $2.66\times10^{-6}$ | Yes | Risk * |
| rs2241894 | *ADH1C* | Silent | T | C | C allele is associated with alcohol dependence | [73] | 4/46 (0.087) | 4724/10016 (0.47) | Probably Damaging / Tolerated | 0.088 | 27.21 | $1.23\times10^{-6}$ | Yes | Resilience |
| rs2298753 | *ADH1C* | 3' UTR | T | C | VUS | - | 13/46 (0.28) | 644/10016 (0.06) | NA/NA | 6.19 | 41.89 | $7.28\times10^{-10}$ | Yes | – * |
| rs1614972 | *ADH1C* | Intron | C | C | T variant is associated with reduced risk of alcohol dependence | [37] | 43/46 (0.93) | 4804/10016 (0.48) | NA / NA | 4.8 | 37.99 | $5.61\times10^{-9}$ | Yes | Risk * |
| rs1042364 | *ADH4* | 3' UTR | T | T | T allele is associated with alcohol dependence | [39] | 8/46 (0.17) | 1174/10016 (0.11) | NA / NA | 6.24 | 4.24 | $1.20\times10^{-1}$ | No | – |
| rs1126671 | *ADH4* | Missense | T | T | T variant affects alcohol binding and protein stability. T allele is assoc. with alcohol dependence | [87] | 11/46 (0.24) | 1532/10016 (0.15) | Probably Damaging / Tolerated | 0.005 | 4.47 | $1.07\times10^{-1}$ | No | Resilience |
| rs3762894 | *ADH4* | Upstream | T | T | A allele – less alcohol consumption; T variant with alcohol dependence | [39] | 42/46 (0.91) | 6824/10016 (0.68) | NA / NA | 0.39 | 11.65 | $4.00\times10^{-3}$ | Yes | Risk * |
| rs971074 | *ADH7* | Silent | C | T | T allele is associated with heroin addition | [74] | 10/46 (0.22) | 1200/10016 (0.12) | NA / NA | 7.79 | 6.84 | $3.20\times10^{-2}$ | No | Risk |
| rs1154470 | *ADH7* | Upstream | G | A | A variant associated with alcohol use disorders | [88] | 15/46 (0.33) | 2424/10016 (0.24) | NA / NA | 0.72 | 2.48 | $2.90\times10^{-1}$ | No | – |

| | | | | | | | | | | | | | | |
|---|---|---|---|---|---|---|---|---|---|---|---|---|---|---|
| rs4646626 | *ALDH1A2* | Missense | C | T | T variant found in Tetralogy of Fallot patients | [89] | 23/46 (0.50) | 3734/10016 (0.37) | Begin / Tolerated | 17.53 | 2.31 | $3.10\times10^{-1}$ | No | – * |
| rs16939660 | *ALDH1A2* | Silent | T | C | C variant found in patients with congenital heart defects, spina bifida and neural tube defects | [89,90] | 1/46 (0.02) | 110/10016 (0.01) | NA / NA | 10.19 | 43.92 | $2.90\times10^{-10}$ | Yes | Risk |
| rs2073478 | *ALDH1B1* | Missense | G | G | TT genotype has been shown to be protective against drinking | [75] | 19/46 (0.41) | 6060/10016 (0.60) | Probably Damaging / Damaging | 20.8 | 7.15 | $3.00\times10^{-2}$ | No | Resilience |
| rs671 | *ALDH2* | Missense | G | G | A allele – reduced alcohol consumption and flushing; enzyme nearly inactive | [40] | 46/46 (1) | 9658/10016 (0.96) | NA / NA | 33 | 14.24 | $8.10\times10^{-4}$ | Yes | Risk * |
| rs10891338 | *BCO2* | Missense | T | C | C allele has a probably Damaging PolyPhen and damaging CADD score | - | 46/46 (1) | 9720/10016 (0.97) | Probably Damaging / Tolerated | 16.04 | 16.86 | $2.20\times10^{-4}$ | Yes | Risk |
| rs35361223 | *BCO2* | Missense | C | G | G allele has a damaging SIFT prediction | - | 2/46 (0.04) | 184/10016 (0.02) | Benign / Damaging | 7.127 | 68.53 | $1.37\times10^{-6}$ | Yes | Risk * |
| rs61743167 | *CES3* | Missense | C | T | T allele has a damaging SIFT and CADD prediction | - | 1/46 (0.02) | 114/10016 (0.011) | Benign / Damaging | 16.67 | 42.32 | $6.45\times10^{-10}$ | Yes | Risk |
| rs12263200 | *CYP26C1* | Intron | G | G | T allele has a damaging SIFT prediction | - | 27/46 (0.59) | 1224/10016 (0.12) | NA / Damaging | 2.71 | 93.39 | $1.00\times10^{-3}$ | Yes | Risk * |
| rs58993699 | *CYP26C1* | Intron | C | C | T allele has a damaging SIFT and CADD prediction | - | 45/46 (0.96) | 9686/10016 (0.97) | NA / Damaging | 14.23 | 11.86 | $1.00\times10^{-3}$ | Yes | Risk |
| rs55962377 | *DGAT1* | Missense | G | T | T allele has damaging PolyPhen and CADD Score | - | 1/46 (0.02) | 360/10016 (0.036) | Probably Damaging / Tolerated | 18.73 | 12.69 | $2.00\times10^{-3}$ | Yes | Resilience |
| rs148632765 | *FABP4* | Missense | C | G | G allele has damaging PolyPhen and CADD Score | - | 1/46 (0.02) | 4/10016 ($4\times10^{-4}$) | Probably Damaging / Tolerated | 22 | 872.33 | $3.77\times10^{-190}$ | Yes | Risk |
| rs3829462 | *LIPC* | Missense | C | A | A allele has damaging PolyPhen and CADD Score | - | 30/46 (0.65) | 9376/10016 (0.94) | Probably Damaging / Tolerated | 20.6 | 68.69 | $3.30\times10^{-15}$ | Yes | Resilience |
| rs61753150 | *NCOR1* | Missense | T | T | G allele has probably damaging PolyPhen score and damaging SIFT and CADD Score | - | 1/46 (0.02) | 40/10016 ($4\times10^{-3}$) | Probably Damaging / Damaging | 22.3 | 121.8 | $3.55\times10^{-27}$ | Yes | Risk |
| rs2227277 | *NCOR2* | Missense | C | C | T allele has probably damaging PolyPhen score and damaging SIFT and CADD Score | - | 3/46 (0.065) | 574/10016 (0.06) | Probably Damaging / Damaging | 17.1 | 7.29 | $3.00\times10^{-2}$ | No | Resilience |

| | | | | | | | | | | | | | | |
|---|---|---|---|---|---|---|---|---|---|---|---|---|---|---|
| rs2134095 | *RXRG* | Silent | G | A | A allele is associated with lower cholesterol levels and gestational diabetes mellitus | [41] | 33/46 (0.72) | 4796/10316 (0.48) | NA / NA | 5.66 | 12.43 | $2.80 \times 10^{-3}$ | Yes | Risk * |
| rs971756 | *STRA6* | Missense | A | T | T variant found in a patient with Matthew-Woods Syndrome | [42] | 6/46 (0.13) | 546/10016 (0.05) | NA / Damaging | 3.86 | 12.71 | $2.00 \times 10^{-3}$ | Yes | Risk * |
| rs11857410 | *STRA6* | Silent | G | A | A allele found with ano- and microphtalmia; and with Matthew-Wood Syndrome | [43] | 6/46 (0.13) | 618/10016 (0.06) | NA / NA | 8.72 | 10.3 | $5.00 \times 10^{-3}$ | Yes | Risk * |

* Genotype validated by TaqMan
