# Supplementary material for: Risk and Resilience Variants in the Retinoic Acid Metabolic and Developmental Pathways Associated with Risk of FASD Outcomes
Source: Biomolecules. 2024 May 10;14(5):569. doi: 10.3390/biom14050569 (PMC11117505; doi:10.3390/biom14050569)
Supplement: Supplementary file 1 [file biomolecules-14-00569-s001.zip › Supplemental Table S6 Variant Analysis Results of Genes in Retinoic Acid-Regulated Pathways - Revised.pdf]

Supplemental Table S6. Variant Analysis Results of Genes in Retinoic Acid-Regulated Pathways.

rsID	Gene	Type of Mutation	Ref Allele	Presump-tive Risk Allele	Biological Impact of Variant	Ref.	FASD Cohort Risk Allele Count Frequency	Thousand Genomes Risk Allele Count (Frequency)	PolyPhen / Sift Prediction	CADD PHRED Score	Statistic	*p*-Value	Passed Benjamini-Hochberg Threshold	Risk or Resilience
rs1805105	*AXIN1**	Silent	A	G	G allele associated with cryptorchidism, congenital heart disease and dilated cardiomyopathy	[52,91,92]	21/46 (0.46)	6400/10016 (0.64)	NA / NA	0.04	6.78	3.30×10^{-2}	No	Resilience
rs117208012	*AXIN1**	Missense	C	T	T allele associated with spinal bone mineral density	[93]	1/46 (0.02)	90/10016 (9×10^{-3})	Benign / Tolerated	15.08	54.03	1.85×10^{-12}	Yes	Risk
rs1133683	*AXIN2**	Silent	G	AA Genotype	AA genotype was associated with increased risk of non-syndromic cleft lip and palate	[44]	40/46 (0.87) *genotype count and frequency shown	5548/10016 (0.55) *genotype count and frequency shown	NA / NA	5.66	33.23	6.09×10^{-8}	Yes	Risk
rs2240308	*AXIN2**	Missense	G	A	A variant associated with cryptorchidism, congenital heart disease and dilated cardiomyopathy	[52,91,92]	30/46 (0.65)	3382/10016 (0.34)	Probably Damaging / Tolerated	17.48	20.44	3.64×10^{-5}	Yes	Risk
rs35415678	*AXIN2**	Silent	G	A	A variant disrupts exonic splicing enhancer sequences and potentially contributes to tooth agenesis	[94]	1/46 (0.02)	1646/10016 (0.16)	NA / NA	8.26	8.45	1.50×10^{-3}	Yes	Resilience
rs1049007	*BMP2**	Silent	A	A	A variant is associated with ossification defects in the posterior longitudinal ligament	[57]	22/46 (0.47)	2544/10016 (0.25)	NA / NA	10.67	12.75	1.70×10^{-3}	Yes	Risk
rs235768	*BMP2**	Silent	A	T	AT genotype is associated with cleft lip and palate, T allele is associated with mandibular retrognathism and ossification defects	[45,51,57]	26/46 (0.52)	768/10016 (0.77)	Probably Damaging / Damaging	23.4	11.16	4.00×10^{-3}	Yes	Resilience
rs17563	*BMP4**	Missense	A	G	G alleles is associated with non-syndromic cleft lip and palate	[46,47]	23/46 (0.5)	3263/10016 (0.33)	Damaging / Tolerated	19.13	6.6	3.00×10^{-2}	No	Risk
rs28360477	*CAMKK2**	Missense	C	T	T allele has damaging SIFT and CADD scores	-	5/46 (0.11)	176/10016 (0.018)	Benign / Damaging	19.6	47.57	4.67×10^{-11}	Yes	Risk

rs45498702	*CAV1**	3' UTR	C	T	T allele associated with arterial fibrillation	[53]	3/46 (0.07)	414/10016 (0.04)	NA / NA	9.61	11.22	4.00×10^{-3}	Yes	Risk
rs8940	*CAV2***	Missense	C	G	G allele has a probably damaging PolyPhen score and damaging SIFT and CADD Scores	-	15/46 (0.326)	1412/10016 (0.141)	Probably Damaging / Damaging	23.4	14.99	5.54×10^{-4}	Yes	Risk
rs183543237	*DAAM2†*	Missense	C	G	G allele has damaging SIFT and CADD scores	-	1/46 (0.02)	10/10016 (1×10^{-3})	NA / Damaging	24.7	434.46	4.56×10^{-95}	Yes	Risk
rs61748650	*DAAM2†*	Missense	T	C	C allele has damaging SIFT and CADD scores	-	1/46 (0.02)	12/10016 (1.2×10^{-3})	NA / Damaging	21.9	371.91	1.74×10^{-81}	Yes	Risk
rs145496306	*DVL1**	Missense	G	A	A allele has a probably damaging PolyPhen score and damaging CADD score	-	1/46 (0.02)	34/10016 (3.4×10^{-3})	Probably Damaging / Tolerated	15.56	142.63	1.07×10^{-31}	Yes	Risk
rs117262744	*DVL2**	Missense	T	C	C variant found in fetal tissue with neural tube defects	[95]	1/46 (0.02)	64/10016 (6.4×10^{-3})	Benign / Tolerated	19.2	76.42	2.55×10^{-17}	Yes	Risk
rs72839768	*DVL2**	Missense	G	A	A allele has damaging PolyPhen, SIFT and CADD scores	-	1/46 (0.02)	72/10016 (7.2×10^{-3})	Damaging / Damaging	26.6	67.85	1.85×10^{-15}	Yes	Risk
rs1140475	*EGFR**	Silent	T	C	Variant located in protein kinase domain, C allele associated with temporomandibular disorder	[64,65]	37/46 (0.80)	9182/10016 (0.92)	NA / NA	0.48	12.04	2.00×10^{-3}	Yes	Resilience
rs61735303	*FZD4**	Missense	G	A	A allele associated with familial exudative vitreoretinopathy and retinopathy of prematurity	[96]	1/46 (0.02)	148/10016 (0.015)	Probably Damaging / Tolerated	24.1	32.21	1.01×10^{-7}	Yes	Risk
rs61735304	*FZD4**	Missense	G	A	A allele has a probably damaging polyphen prediction and damaging CADD score	-	1/46 (0.02)	126/10016 (0.011)	Probably Damaging / Tolerated	16.33	38.13	5.25×10^{-9}	Yes	Risk
rs61749246	*FZD4**	3' UTR	C	A	A allele associated with retinopathy of prematurity	[97]	3/46 (0.06)	108/10016 (0.01)	NA / NA	1.44	55.77	7.75×10^{-13}	Yes	Risk
rs12549394	*FZD6**	Missense	C	A	A allele found in patients with neural tube defects	[95]	7/46 (0.15)	174/10016 (0.02)	Benign / Damaging	0.052	73.02	1.39×10^{-16}	Yes	Risk
rs201094355	*FZD7†*	Missense	G	T	T allele has probably damaging PolyPhen score and damaging SIFT and CADD scores	-	2/46 (0.04)	2/10016 (2×10^{-4})	Probably Damaging / Damaging	34	1177.82	1.74×10^{-256}	Yes	Risk
rs2228226	*GLI1†*	Missense	G	C	C variant results in 50% transactivation activity in vitro, congenital heart defects and hypospadias	[56,98,99]	18/46 (0.39)	5872/10016 (0.59)	Benign / Tolerated	18.95	7.23	3.00×10^{-2}	No	Resilience

rs150417879	*GLI2**	Missense	G	A	A allele has probably damaging PolyPhen score and damaging SIFT and CADD scores	-	1/46 (0.02)	2/10016 (2×10^{-4})	Probably Damaging / Damaging	18.73	1310.22	3.10×10^{-285}	Yes	Resilience
rs2592595	*GLI2**	Silent	G	A	A variant found in study of ectrodactyly patients	[61]	46/46 (1)	8286/10016 (0.83)	NA / Tolerated	0.004	11.1	4.00×10^{-3}	Yes	Risk
rs35280470	*GLI3**	Missense	C	T	T allele has a damaging Polyphen, SIFT and CADD scores	-	2/46 (0.04)	152/10016 (0.015)	Damaging / Damaging	17.71	33.4	5.60×10^{-8}	Yes	Resilience
rs35364414	*GLI3*	Missense	G	A	A allele is associated with NTD	[100]	2/46 (0.04)	192/10016 (0.019)	Damaging / Damaging	28.9	25.74	2.60×10^{-6}	Yes	Risk
rs121917710	*GLI3*	Missense	C	T	T allele PolyPhen, SIFT and CADD predictions are damaging, and also found in ASD individuals	[101]	1/46 (0.02)	26/10016 (3×10^{-3})	Probably Damaging / Damaging	27.4	184.3	9.60×10^{-41}	Yes	Risk
rs45571645	*HOXA1**	Missense	G	T	T allele has probably Damaging PolyPhen and damaging CADD score	-	1/46 (0.02)	54/10016 (5.4×10^{-3})	Probably Damaging / Tolerated	23.5	90.59	2.13×10^{-20}	Yes	Risk
rs35115415	*HOXB1**	Silent	C	T	T allele found in ASD patients	[102]	1/46 (0.02)	20/10016 (2×10^{-3})	NA / NA	11.53	235.46	7.44×10^{-52}	Yes	Risk
rs61751489	*NOTCH1**	Missense	C	T	T allele associated with bicuspid aortic valve congenital defects	[103]	6/46 (0.13)	212/10016 (0.02)	Benign / Tolerated	7.42	47.18	5.70×10^{-11}	Yes	Risk
rs76371972	*NOTCH1**	Missense	C	T	T allele with bicuspid aortic valve and aortopathy	[104]	2/46 (0.04)	70/10016 (7×10^{-3})	Probably Damaging / Tolerated	23.8	75.92	3.28×10^{-17}	Yes	Risk
rs61751542	*NOTCH1**	Missense	G	A	A allele is associated bicuspid aortic valve and aortopathy	[104]	1/46 (0.02)	64/10016 (6×10^{-3})	Benign / Tolerated	4.04	76.42	2.55×10^{-17}	Yes	Risk
rs1044006	*NOTCH3**	Silent	T	C	C allele is associated with cerebral infarction and mandibular prognathism	[48,105]	33/46 (0.71)	8732/10016 (0.87)	NA / NA	0.17	12.17	2.00×10^{-3}	Yes	Resilience
rs10408676	*NOTCH3**	Missense	C	T	T variant has a damaging PolyPhen and CADD prediction	-	1/46 (0.02)	846/10016 (0.085)	Damaging / Tolerated	23.5	6.82	3.30×10^{-2}	No	Resilience
rs35769976	*NOTCH3**	Missense	C	G	G allele leads to a significant alternation to protein structure	[63]	1/46 (0.02)	1104/10016 (0.11)	Benign / Tolerated	14.94	6.78	3.30×10^{-2}	No	Resilience
rs8192591	*NOTCH4**	Missense	C	T	T allele has damaging SIFT and CADD Prediction	-	1/46 (0.02)	312/10016 (0.0312)	Benign / Damaging	14.7	14.68	6.50×10^{-4}	Yes	Resilience
rs118133093	*PI4KA†*	Missense	C	G	G allele has damaging SIFT and CADD prediction	-	1/46 (0.02)	128/10016 (0.0128)	NA / NA	12.82	37.51	7.16×10^{-9}	Yes	Risk

rs139747674	*PRICKLE2†*	Missense	C	T	T variant is associated with ASD and partial loss of protein function and dendritic arborisation	[106]	1/46 (0.02)	16/10016 (0.016)	Benign / Tolerated	19.3	288.52	2.24×10^{-63}	Yes	Risk
rs2066836	*PTCH1**	Silent	G	G	A allele is associated with reduced risk of non-syndromic cleft lip and palate	[49]	15/46 (0.33)	920/10016 (0.092)	NA / NA	0.05	33.71	4.80×10^{-8}	Yes	Resilience
rs11573590	*PTCH2**	Missense	G	A	A allele has damaging PolyPhen, SIFT and CADD score	-	2/46 (0.04)	630/10016 (0.0634)	Damaging / Damaging	21.5	6.78	3.40×10^{-2}	No	Resilience
rs1638630	*PTCHD3†*	Missense	T	C	C allele has a probably damaging polyphen score	-	2/46 (0.04)	9766/10016 (0.98)	Probably Damaging / NA	5.26	19.05	7.31×10^{-5}	Yes	Resilience
rs149901958	*RHOD*	Missense	C	T	T allele has damaging PolyPhen, SIFT and CADD predictions	-	5/46 (0.11)	26/10016 (2.6×10^{-3})	Damaging / Damaging	24.3	318.41	7.20×10^{-70}	Yes	Risk
rs35364374	*RYR1**	Missense	G	T	T allele has damaging PolyPhe and CADD scores	-	1/46 (0.02)	550/10016 (0.055)	Damaging / Tolerated	19.49	8.62	1.30×10^{-2}	Yes	Resilience
rs145044872	*RYR1**	Missense	G	A	A allele has probably damaging PolyPhen score and damaging SIFT and CADD score	-	1/46 (0.02)	8/10016 (8×10^{-4})	Probably Damaging / Damaging	29.6	522.03	4.40×10^{-114}	Yes	Resilience
rs41315858	*RYR2**	Missense	G	A	A allele results in increased Ca^{2+} oscillation in response to caffeine and associated with arrhythmogenic right ventricular cardiomyopathy	[54,107]	1/46 (0.02)	98/10016 (0.01)	NA / Tolerated	0.12	49.5	1.78×10^{-11}	Yes	Risk
rs3766871	*RYR2**	Missense	G	A	The A allele associated with channel destabilization, increased intracellular Ca^{2+} oscillation, cardiac arrhythmias and ventricular tachycardia	[54]	1/46 (0.02)	765/10016 (0.08)	NA / Tolerated	13.62	7.05	2.90×10^{-2}	No	Resilience
rs1802074	*SFRP4**	Missense	C	T	T allele is associated with failing hearts	[55]	4/46 (0.09)	2534/10016 (0.25)	Benign / Tolerated	2.1	7.34	2.50×10^{-2}	No	Resilience
rs143647630	*SFRP4**	Missense	C	T	T allele has probably Damaging PolyPhen and damaging CADD score	-	1/46 (0.02)	10/10016 (1×10^{-3})	Probably Damaging / Tolerated	29.8	434.46	4.56×10^{-95}	Yes	Risk
rs139884	*SOX10**	Silent	A	G	G allele found in patients of Waardenburg Type I and II Syndromes	[50]	21/46 (0.46)	7144/10016 (0.71)	NA / NA	0.28	15.16	5.10×10^{-4}	Yes	Resilience
rs61758378	*WNT1**	Missense	T	A	A allele results in an overactive protein, and found in ASD patients	[108]	1/46 (0.02)	28/10016 (3×10^{-3})	Benign / Tolerated	16.9	171.8	4.95×10^{-38}	Yes	Risk
rs1051886	*WNT10B**	Silent	G	A	A allele is associated with hip BMD	[58]	24/46 (0.52)	3116/10016 (0.31)	NA / NA	28.1	9.79	7.00×10^{-3}	Yes	Risk

rs35034312	*WNT10B**	Missense	G	G	G allele stabilizes the beta-catenin complex	[109]	45/46 (0.98)	9980/10016 (0.99)	Probably Damaging / Tolerated	9.79	134.96	4.95×10^{-30}	Yes	Resilience
rs121908120	*WNT10A†*	Missense	T	A	A allele associated with Odontoonychodermal dysplasia	[110]	1/46 (0.02)	60/10016 (6×10^{-3})	Damaging / Damaging	21.3	81.54	1.97×10^{-18}	Yes	Risk
rs2908004	*WNT16†*	Missense	G	G	G allele increases gene expression and associated with increased risk of fracture	[59,111]	31/46 (0.67)	4904/10016 (0.49)	Benign / Tolerated	15.08	6.22	4.50×10^{-2}	No	Risk
rs2707466	*WNT16†*	Missense	C	C	C allele is associated with lower BMD and fractures	[60]	32/46 (0.70)	4978/10016 (0.49)	Benign / Tolerated	16.8	7.23	2.70×10^{-2}	No	Risk
rs142980721	*WNT2B**	Missense	G	A	A allele has a probably damaging polyphen score and damaging SIFT score	-	2/46 (0.04)	210/10016 (0.021)	Probably Damaging / Damaging	2.13	23.26	8.09×10^{-4}	Yes	Risk
rs41270173	*WNT3A**	3’ UTR	G	A	A allele has damaging SIFT and CADD scores	-	1/46 (0.02)	16/10016 (1.6×10^{-3})	NA / Damaging	13.3	288.52	2.24×10^{-63}	Yes	Risk
rs199755858	*WNT8A**	Missense	C	G	G allele has damaging SIFT and CADD scores	-	1/46 (0.02)	26/10016 (3.6×10^{-3})	Benign / Damaging	11.95	184.3	9.55×10^{-41}	Yes	Risk
rs34072914	*WNT9B*	Silent	G	T	T allele is associated with Mayer-Rokitansky-Kuster-Hauser Syndrome	[62]	1/46 (0.02)	330/10016 (0.03)	NA / NA	12.75	13.86	9.00×10^{-4}	Yes	Resilience

* Directly regulated by retinoic acid

† Upstream or downstream of retinoic acid-controlled pathways
