# Supplementary material for: Risk and Resilience Variants in the Retinoic Acid Metabolic and Developmental Pathways Associated with Risk of FASD Outcomes
Source: Biomolecules. 2024 May 10;14(5):569. doi: 10.3390/biom14050569 (PMC11117505; doi:10.3390/biom14050569)
Supplement: Supplementary file 1 [file biomolecules-14-00569-s001.zip › Supplemental Table S7 Variant Analysis Results of the Causative Genes of Rare Neurodevelopmental Disorders - revised.pdf]

Supplemental Table S7. Variant Analysis Results of the Causative Genes of Rare Neurodevelopmental Disorders.

rsID	Gene	Type of Mutation	Ref Allele	Presump-tive Risk Allele	Biological Impact of Variant	Ref.	FASD Cohort Risk Allele Count Frequency	Thousand Genomes Risk Allele Count (Frequency)	PolyPhen / Sift Prediction	CADD PHRED Score	Statistic	p-Value	Passed Benjamini -Hochberg Threshold	Risk or Resilience
rs61921916	*A2ML1*	Missense	C	A	A allele has damaging CADD and PolyPhen prediction	-	1/46 (0.02)	30/10016 (3×10^{-3})	Damaging / Tolerated	19.55	160.86	1.18×10^{-35}	Yes	Risk
rs2230808	*ABCA1*	Missense	T	C	C allele associated with more severe SLOS phenotypes in SLOS patients	[69]	35/46 (0.76)	5392/10016 (0.58)	Benign / Tolerated	22	9.14	1.00×10^{-2}	Yes	Risk
rs145694621	*ANKRD11†*	Missense	G	C	C allele has damaging Polyphen and SIFT score	-	1/46 (0.02)	50/10016 (5×10^{-3})	Benign / Damaging	0.13	110.94	8.11×10^{-25}	Yes	Risk
rs80068543	*ARVCF**	Missense	C	T	T allele associated with Hirschsprung's Disease	[112]	1/46 (0.02)	368/10016 (0.04)	Benign / Tolerated	17.93	12.41	2.00×10^{-3}	Yes	Resilience
rs115344498	*ARVCF**	Missense	A	G	G allele has damaging SIFT and CADD Score	-	1/46 (0.02)	6/10016 (6×10^{-4})	Benign / Damaging	22	653.39	1.31×10^{-142}	Yes	Risk
rs35676845	*BRD4**	Missense	C	T	T allele has probably damaging PolyPhen score and damaging SIFT and CADD Score	-	1/46 (0.02)	62/10016 (6×10^{-3})	Probably Damaging / Damaging	26.7	78.9	7.38×10^{-18}	Yes	Risk
rs3208856	*CBLC**	Missense	C	T	T allele has damaging PolyPhen, SIFTand CADD scores	-	4/46 (0.087)	194/10016 (0.02)	Damaging / Damaging	15.71	34.6	3.08×10^{-8}	Yes	Risk
rs11231887	*CDC42BPG**	Missense	C	T	T allele has a damaging Polyphenand CADD score	-	1/46 (0.02)	190/10016 (0.019)	Damaging / Tolerated	24.2	24.74	4.24×10^{-6}	Yes	Risk
rs55975541	*CDC42BPG**	Missense	G	A	A allele has damaging PolyPhen, SIFT and CADD predictions	-	5/46 (0.11)	296/10016 (0.03)	Damaging / Damaging	28.9	25.03	3.66×10^{-6}	Yes	Risk
rs41272435	*CHD7‡*	Missense	T	A	A allele has damaging SIFT and CADD predictions	-	3/46 (0.065)	52/10016 (5.2×10^{-3})	NA / Damaging	22.5	118.19	2.16×10^{-26}	Yes	Risk
rs186499367	*CHD7‡*	Missense	G	T	G allele has damaging SIFT and CADD predictions	-	1/46 (0.02)	2/10016 (2×10^{-4})	NA / Damaging	25	1310.22	3.08×10^{-285}	Yes	Risk
rs199828744	*CHD7‡*	Silent	C	G	G allele found in two CHARGE syndrome patients	[113]	1/46 (0.02)	2/10016 (2×10^{-4})	NA / NA	0.53	1310.22	3.10×10^{-285}	Yes	Risk

rs712952	*CLTCL1†*	Missense	G	A	A allele has a damaging SIFT and CADD scores	-	5/46 (0.11)	510/10016 (0.0509)	NA / Damaging	15.88	11.42	3.30×10^{-3}	Yes	Risk
rs807459	*CLTCL1†*	Missense	T	C	C allele has a damaging SIFT and CADD scores	-	5/46 (0.11)	552/10016 (0.0551)	NA / Damaging	15.88	10.06	6.50×10^{-3}	Yes	Risk
rs147685377	*CLTCL1†*	Missense	T	C	C allele has a damaging SIFT and CADD predictions	-	1/46 (0.02)	6/10016 (6×10^{-4})	NA / Damaging	20.8	653.39	1.32×10^{-142}	Yes	Risk
rs190351859	*CLTCL1†*	Missense	G	A	A allele has a damaging PolyPhen and CADD predictions	-	2/46 (0.04)	64/10016 (6×10^{-3})	NA / Damaging	22.5	83.15	8.80×10^{-19}	Yes	Risk
rs769224	*COMT*	Silent	G	G	G allele associated with heroin usage	[114]	44/46 (0/96)	9512/10016 (0.95)	NA / NA	2.57	8.5	1.40×10^{-2}	Yes	Risk
rs61732148	*CUL7†*	Missense	G	A	A allele has damaging SIFT and CADD predictions	-	2/46 (0.04)	172/10016 (0.0172)	Benign / Damaging	21.1	29.12	4.75×10^{-7}	Yes	Risk
rs138659167	*DHCR7^Δ*	Missense	C	G	G allele is a pathogenic variant for SLOS, homozygosity leads to a severe SLOS phenotype or early miscarriage	[71]	2/46 (0.04)	26/10016 (3×10^{-3})	NA / NA	34	198.69	7.17×10^{-44}	Yes	Risk
rs75659311	*FGD1†*	Missense	G	A	A allele has a probably damaging PolyPhen score and damaging SIFT Score	-	2/46 (0.04)	180/10016 (0.0238)	Probably Damaging / Damaging	8.42	20.03	4.48×10^{-5}	Yes	Risk
rs1047057	*FGFR2**	Silent	G	A	A allele found in a patient in Apert's Syndrome	[67]	30/46 (0.65)	4150/10016 (0.41)	NA / NA	18.4	10.73	5.00×10^{-3}	Yes	Risk
rs3750819	*FGFR2**	Missense	C	A	A allele has damaging SIFT and CADD predictions	-	1/46 (0.02)	126/10016 (0.0126)	Probably Damaging / Tolerated	20.1	87.36	1.10×10^{-19}	Yes	Risk
rs73148914	*GNB1L*	Missense	G	A	A allele has probably damaging PolyPhen score and damaging SIFT and CADD Scores	-	1/46 (0.02)	60/10016 (6×10^{-3})	Probably Damaging / Damaging	21.1	81.54	1.97×10^{-18}	Yes	Risk
rs2287256	*KLHL22*	3' UTR	C	T	T allele has a damaging SIFT score	-	1/46 (0.02)	140/10016 (0.014)	NA / Damaging	7.1	34.15	3.84×10^{-8}	Yes	Risk
rs80230662	*HIC2*	Missense	C	T	T allele has probably damaging PolyPhen score and damaging CADD score	-	2/46 (0.04)	214/10016 (0.0214)	Damaging / Tolerated	19.69	22.77	1.14×10^{-5}	Yes	Risk
rs201035504	*MED15*	Missense	C	A	A allele has probably damaging PolyPhen score and damaging SIFT and CADD Scores	-	2/46 (0.04)	6/10016 (6×10^{-4})	Probably Damaging / Damaging	25	651.95	2.70×10^{-142}	Yes	Risk
rs147106995	*MID1†*	Missense	G	A	A allele has damaging PolyPhen, SIFT and CADD Scores	-	1/46 (0.02)	64/10016 (8.5×10^{-3})	Damaging / Damaging	23.9	57.01	4.17×10^{-13}	Yes	Risk

rs10932816	*OBSL1*	Silent	A	G	G allele has a damaging SIFT prediction	-	46/46 (1)	9652/10016 (0.9637)	NA / Damaging	0.17	14.04	8.90×10^{-4}	Yes	Risk
rs147763320	*OBSL1*	Missense	G	T	T allele has a damaging SIFT and CADD predictions	-	1/46 (0.02)	2/10016 (2×10^{-4})	NA / Damaging	24.8	1310.22	3.08×10^{-285}	Yes	Risk
rs4819756	*PRODH*	Missense	A	A	A allele results in 30-70% drop in enzymatic activity and associated with Schizophrenia	[115, 116]	25/46 (0.54)	2194/10016 (0.22)	Probably Damaging / Tolerated	8.57	28.94	5.20×10^{-7}	Yes	Risk
rs2277838	*P2RX6*	Missense	G	A	A allele has a damaging SIFT score	-	6/46 (0.13)	780/10016 (0.0779)	NA / Damaging	23	6.69	3.50×10^{-2}	No	Resilience
rs2227902	*REST**	Missense	G	T	T allele is associated with hippocampal volume loss and impairment in memory	[117, 118]	4/46 (0.087)	646/10016 (0.065)	Probably Damaging / Tolerated	6.29	6.65	3.60×10^{-2}	No	Risk
rs61748756	*REST**	Missense	C	G	G allele has damaging SIFT prediction	-	2/46 (0.04)	60/10016 (6×10^{-3})	Benign / Damaging	0.16	88.74	5.38×10^{-20}	Yes	Risk
rs361566	*SCARF2*	Missense	G	A	A allele has a damaging PolyPhen and CADD scores	-	9/46 (0.195)	1126/10016 (0.1124)	Damaging / Tolerated	27.3	6.16	4.60×10^{-2}	No	Risk
rs874100	*SCARF2*	Missense	G	C	C allele has a probably damaging PolyPhen score and damaging SIFT and CADD scores	-	2/46 (0.04)	2426/10016 (0.2422)	Probably Damaging / Damaging	11.35	10.61	5.00×10^{-3}	Yes	Resilience
rs874101	*SCARF2*	Missense	G	C	C allele has a probably damaging PolyPhen score	-	15/46 (0.33)	5354/10016 (0.5345)	Probably Damaging / Tolerated	7.37	8	1.80×10^{-2}	Yes	Resilience
rs116892729	*SNAP29*	Missense	A	G	G allele has a damaging SIFT and CADD scores	-	1/46 (0.02)	140/10016 (0.014)	Benign / Damaging	24.8	34.15	3.84×10^{-8}	Yes	Risk
rs2229989	*SOX9**	Silent	C	T	G and A allele are associated with reduced transactivation capacity of the SOX9 protein and are associated with mild campomelic dysplasia	[66]	11/46 (0.24)	1368/10016 (0.14)	NA / NA	11.83	6.3	4.00×10^{-2}	No	Risk
rs41298840	*TBX1**	Silent	G	A	A allele is enriched in 22q11.2DS children	[119]	42/46 (0.91)	7706/10016 (0.77)	NA / NA	11.64	6.16	4.50×10^{-2}	No	Risk
rs5746826	*TBX1**	3' UTR	A	T	T allele is associated with poor executive functioning in patients with schizophrenia	[120]	14/46 (0.30)	5936/10016 (0.59)	NA / NA	0.25	15.82	4.00×10^{-4}	Yes	Resilience
rs3747052	*TSSK2*	Missense	A	G	G allele has a damaging SIFT and CADD scores	-	1/46 (0.02)	392/10016 (0.0391)	Benign / Damaging	11.22	11.66	2.90×10^{-3}	Yes	Resilience
rs148092370	*TXNRD2*	Missense	T	A	A allele has a damaging Polyphen and damaging SIFT score	-	1/46 (0.02)	12/10016 (1.2×10^{-3})	Damaging / Damaging	8.55	371.91	1.74×10^{-81}	Yes	Risk

rs2871053	*ZDHHC8*	Upstream	T	C	C allele has a damaging SIFT score	-	3/46 (0.07)	506/10016 (0.0505)	NA / Damaging	3.51	8.6	1.40×10^{-2}	Yes	Risk

*Directly regulated by retinoic acid

†Upstream or downstream of retinoic acid-controlled pathways

‡Regulates RA metabolism genes

Δ Genotype validated by TaqMan
