# Supplementary material for: Risk and Resilience Variants in the Retinoic Acid Metabolic and Developmental Pathways Associated with Risk of FASD Outcomes
Source: Biomolecules. 2024 May 10;14(5):569. doi: 10.3390/biom14050569 (PMC11117505; doi:10.3390/biom14050569)
Supplement: Supplementary file 1 [file biomolecules-14-00569-s001.zip › Supplemental Table S8 Variant Analysis Results of Neurotransmitter, Forebrain Development and Collagen Genes - revised.pdf]

Supplemental Table S8. Variant Analysis Results of Neurotransmitter, Forebrain Development and Collagen Genes.

rsID	Gene	Type of Mutation	Ref Allele	Presump-tive Risk Allele	Biological Impact of Variant	Ref.	FASD Cohort Risk Allele Count Frequency	Thousand Genomes Risk Allele Count (Frequency)	PolyPhen / Sift Prediction	CADD PHRED Score	Statistic	p-Value	Passed Benjamini-Hochberg Threshold	Risk or Resilience
rs145379118	*ARNT2*	Missense	G	T	T allele has Probably damaging PolyPhen and damaging SIFT and CADD scores	-	1/46 (0.02)	14/10016 (1.4×10^{-3})	Probably Damaging / Damaging	23.3	325	2.67×10^{-71}	Yes	Risk
rs1064583	*COL10A1**	Missense	A	G	G allele predicted to affect protein structure and splicing	[121]	12/46 (0.26)	44462/10016 (0.44)	Benign / Tolerated	12.91	6.34	4.00×10^{-2}	No	Resilience
rs200235459	*COL10A1**	Missense	C	G	G allele has damaging PolyPhen and CADD predictions	-	6/46 (0.13)	2/10016 (2×10^{-4})	Damaging / Tolerated	19.07	1426.96	1.38×10^{-310}	Yes	Risk
rs1635553	*COL2A1**	Silent	A	G	G allele associated with hip osteoarthritis	[122]	11/46 (0.24)	4836/10016 (0.48)	NA / NA	0.047	10.9	4.00×10^{-3}	Yes	Resilience
rs2070739	*COL2A1**	Missense	C	T	T allele associated with intervertebral disc degeneration and brain and eye congenital toxoplasmosis defects	[123,124]	14/46 (0.30)	1712/10016 (0.17)	Probably Damaging / Damaging	31	7.25	2.60×10^{-2}	No	Risk
rs34392760	*COL2A1**	Missense	T	A	A allele has a probably damaging PolyPhen and damaging CADD score	-	1/46 (0.02)	180/10016 (0.018)	Probably Damaging / Tolerated	19.11	26.2	2.10×10^{-6}	Yes	Risk
rs6271	*DBH**	Missense	C	T	T allele is predicted to alter protein structure, synthensis and activity, and associated with bipolar disorder, schizophrenia and IBD	[125-129]	1/46 (0.02)	208/10016 (0.02)	Probably Damaging / Tolerated	19.21	22.5	1.31×10^{-5}	Yes	Risk
rs4531	*DBH**	Missense	G	T	T allele associated with IBD and decreases in enzyme secretion and activity	[126,127]	2/46 (0.04)	654/10016 (0.07)	Benign / Tolerated	1.27	6.55	4.00×10^{-2}	No	Resilience
rs6275	*DRD2**	Silent	A	A	A allele is associated with a reduction in DRD2 expression, alcohol use disorder and disk displacement	[130-132]	11/46 (0.24)	4724/10016 (0.47)	NA / NA	0.036	10.09	6.00×10^{-3}	Yes	Resilience
rs936462	*DRD4*	Upstream	G	A	A allele is associated with heroin addiction	[133]	20/46 (0.43)	8906/10016 (0.88)	NA / NA	2.94	97.33	7.32×10^{-22}	Yes	Resilience

rs747302	*DRD4*	Upstream	G	C	C allele is associated reduced expression, ADHD, schizophrenia and heroin/opiate dependence and addiction	[134-138]	1/46 (0.02)	4580/10016 (0.45)	NA / NA	6.77	35.04	2.46×10^{-8}	Yes	Resilience
rs199815231	*DRD4*	Missense	G	A	A allele has damaging PolyPhen, SIFT and CADD scores	-	1/46 (0.02)	4/10016 (4×10^{-4})	Damaging / Damaging	24.4	872.33	3.77×10^{-190}	Yes	Risk
rs1800044	*HTR1A**	Missense	C	A	A allele assoiciated with loss of signal transduction, major depression and Tourette's Syndrome	[139-140]	1/46 (0.02)	28/10016 (2×10^{-3})	Damaging / Tolerated	23.6	171.8	4.95×10^{-38}	Yes	Risk
rs6303	*HTR1E*	Missense	C	T	T allele has damaging PolyPhen and CADD score	-	1/46 (0.02)	10/10016 (1×10^{-3})	Damaging / Tolerated	23.1	434.46	4.56×10^{-95}	Yes	Risk
rs3125	*HTR2A*	3'UTR	C	G	G allele associated with schizophrenia and depression	[141-143]	11/46 (0.24)	1262/10016 (0.13)	NA / NA	0.1	7.82	2.00×10^{-2}	Yes	Risk
rs6313	*HTR2A*	Silent	G	G	G allele associated with reduced expression. poor mental health, OCD, schizophrenia, alcohol consumption and relapse	[144-148]	41/46 (0.89)	5596/10016 (0.56)	NA / NA	6.52	20.59	3.38×10^{-5}	Yes	Risk
rs17116138	*HTR3B**	Missense	G	A	A allele results in less expression of HTR3B	[149]	2/46 (0.04)	454/10016 (0.045)	Benign / Tolerated	13.17	9.54	8.50×10^{-3}	Yes	Resilience
rs73183412	*HTR3D*	Missense	C	G	G allele has damaging PolyPhen, SIFT and CADD scores	-	5/46 (0.11)	614/10016 (0.0613)	Damaging / Damaging	16.96	8.43	1.50×10^{-2}	Yes	Risk
rs1800883	*HTR5A**	3' UTR	G	G	G allele associated with bipolar disorder, depression, schizophrenia and borderline personality disorder	[150-152]	35/46 (0.76)	5486/10016 (0.54)	NA / NA	6.91	8.42	1.40×10^{-2}	Yes	Risk

*Directly regulated by retinoic acid
