# Supplementary material for: Risk and Resilience Variants in the Retinoic Acid Metabolic and Developmental Pathways Associated with Risk of FASD Outcomes
Source: Biomolecules. 2024 May 10;14(5):569. doi: 10.3390/biom14050569 (PMC11117505; doi:10.3390/biom14050569)
Supplement: Supplementary file 1 [file biomolecules-14-00569-s001.zip › Supplemental Table S9 Variants in Genes Found by FASD Diagnosis.pdf]

Supplemental Table S9. Variants in Genes Found by FASD Diagnosis.

Genes Shared Between FAS and pFAS*	Genes Unique to FAS	Genes Unique to pFAS	Genes Unique to ARND	Genes Shared Between FAS, pFAS, and ARND†
DHCR7, FZD4, HTR3D‡, *PTCH1, PTCH2, SFRP4, SOX9, STRA6*	*ARNT2, DAAM2, FABP4, GNB1L, KLHL22, WNT1 WNT3A, WNT8A*	*A2ML1, ALDH1A2, ANKRD11, BRD4, CES3, CDC42BPG, CHD7, DGAT1, DVL1, DVL2, HIC2, HOXA1, HOXB1, HTR1A, HTR3B, MED15, MID1, PI4KA, PRICKLE2, SNAP29, TSSK2, TXNRD2, WNT10A, WNT2B, WNT9B, ZDHHC8*	*HTR1E*	*ABCA1, ADH1B, ADH1C, ADH4, ADH7 ALDH1B1, ALDH2, AXIN1, AXIN2, BCO2, BMP2, BMP4, CAMKK2, CAV2, CLTCL1, COL10A1, COL1A2, COMT, CYP26C1, DRD2, DRD4, EGFR, FGFR2, FZD6, GLI1, GLI2, HTR2A, HTR5A, LIPC, NCOR1, NCOR2, NOTCH1, NOTCH3, PRODH, PTCH3, P2RX6, OBSL1, RHOD, RXRG, SCARF2, SOX10, TBX1, WNT10B, WNT16*

*****Genes common to FAS and pFAS diagnoses

†Genes common to FAS, pFAS, and ARND diagnoses

‡Did not find any interactions with developmental pathways assessed
